# Plasma campesterol and *ABCG5*/*ABCG8* gene loci on the risk of cholelithiasis and cholecystitis: evidence from Mendelian randomization and colocalization analyses

**DOI:** 10.1186/s40246-024-00583-y

**Published:** 2024-02-12

**Authors:** Jiarui Mi, Qingwei Jiang, Zhengwei Qi, Zhengye Liu, Xiaoyin Bai, Xia Zheng, Jiaguo Wu, Yanfei Fang, Aiming Yang, Haotian Chen

**Affiliations:** 1 Department of Gastroenterology, Sir Run Run Shaw Hospital, School of Medicine, Zhejiang University, Hangzhou, 310016 China; 2 Department of Gastroenterology, Peking Union Medical College Hospital, Peking Union Medical College and Chinese Academy of Medical Sciences, Beijing, 100730 China; 3 Department of Plastic and Aesthetic Center, The First Affiliated Hospital, School of Medicine, Zhejiang University, Hangzhou, 310003 Zhejiang China

**Keywords:** Cholelithiasis, Cholecystitis, Campesterol, Mendelian randomization, Colocalization analysis, Risk factor, Genome-wide association study

## Abstract

The causal relationships between plasma metabolites and cholelithiasis/cholecystitis risks remain elusive. Using two-sample Mendelian randomization, we found that genetic proxied plasma campesterol level showed negative correlation with the risk of both cholelithiasis and cholecystitis. Furthermore, the increased risk of cholelithiasis is correlating with the increased level of plasma campesterol. Lastly, genetic colocalization study showed that the leading SNP, rs4299376, which residing at the ABCG5/ABCG8 gene loci, was shared by plasma campesterol level and cholelithiasis, indicating that the aberrant transportation of plant sterol/cholesterol from the blood stream to the bile duct/gut lumen might be the key in preventing cholesterol gallstone formation.

**Supplementary Information:**

The online version contains supplementary material available at 10.1186/s40246-024-00583-y.

## Introduction

Cholelithiasis, also known as gallstone disease, is a common hepatobiliary disorder. The formation of gallstone is the major risk factor for developing cholecystitis [1]. Cholelithiasis can be primarily subdivided into cholesterol gallstone and pigment gallstones, among which, the cholesterol gallstone is much more commonly seen. It is accepted that the formation of cholesterol gallstones is related to the factors such as the hyper-saturation and crystallization of cholesterol in the bile and the decrease of gallbladder contractility.

Accumulating evidence has shown that modifiable factors were potential risk factors leading to the increased risk of cholesterol gallstone [2]. Moreover, the genome-wide association studies (GWASs) have identified multiple genetic variants associated with increased the genetic predisposition of cholelithiasis in subpopulation. The mendelian randomization (MR) study was considered as an effective method in identifying the causal inference between the exposure and the outcome. Using genetic variants as proxies for gene expression, protein levels and plasma metabolites, researchers are now able to uncover the underlying molecular mechanism of disease pathogenesis and consequently identify new therapeutic targets for drug discovery or drug repurposing.

To date, we, and other researchers, have been employing the summary-level data from these studies to interrogate the causal inferences of multiple metabolic traits on the risk of various disorders. As for cholelithiasis, our study highlighted that genetically predicted higher levels of sphingomyelin and a higher degree of unsaturation of fatty acid were potential protective factors in reducing the risk of cholelithiasis [3]. However, due to the lack of metabolic quantitative trait locus (mQTL) studies on specific metabolites, we were uncapable to generalize the function of a specific metabolic factor on the risk prediction and pathogenesis of cholelithiasis.

Here, we employed summary-level results from the most recent large-scale mQTL study and identified that the decreased plasma campesterol was potentially associated with reducing the risk of cholelithiasis and cholecystitis. Genetic colocalization study identify one SNP locating at the *ABCG5*/*ABCG8* locus is shared by both campesterol and cholelithiasis, indicating the potential therapeutic target in preventing cholesterol gallstone formation.

## Materials and methods

### Metabolic profiles and the GWAS from CLSA cohort

The metabolite measurements for the MR analyses were derived from the 1,458 human metabolites quantified in plasma samples using the ultrahigh performance liquid chromatography–tandem mass spectroscopy platform [4]. This study involved 8,299 random-selected, unrelated, European ancestry individuals from the Canadian Longitudinal Study of Aging (CLSA) cohort. For the follow-up GWAS, the metabolite levels were natural log-transformed with further removal of outliers that are three standard deviations away, and then scaled and normalized. The criteria used for quality control and details for the association testing were described in the original article.

### Selection of instrumental variables

To satisfy the strict criteria, based on the three major MR assumptions, for the mitigation of the horizontal pleiotropy, only the independent genome-wide significant SNPs associated with the metabolites were employed as instrumental variables for the exposure. SNP on FADS locus and extended major histocompatibility complex region on chromosome 6 (± 500 kb) were removed due to strong pleiotropy. The detailed information of SNPs is summarized in by the original study [4].

### Data sources of outcomes

We used the cholelithiasis and cholecystitis related traits GWAS summary statistics from FinnGen Round 9 (https://r9.finngen.fi/) and UK Biobank for two-sample MR. The details of cholelithiasis in FinnGen R9 is summarized as “The presence of calculi in the gallbladder” with corresponding ICD number (ICD-10:K80, ICD-9:574, ICD-8:574). The details of cholecystitis in FinnGen R9 is summarized with the corresponding ICD number (ICD-10:K81, ICD-9:575, ICD-8:5750). This GWAS includes 37,041 cases and 330,903 controls. 20,175,454 SNPs (with the refence build GRCh38). The outcome datasets from UK Biobank, including cholelithiasis (7,426 cases and 448,922 controls), cholelithiasis with acute cholecystitis (1,559 cases and 454,789 controls), cholelithiasis with other cholecystitis (5,570 cases, 450,778 controls), cholecystitis without cholelithiasis (2,650 cases and 453,698 controls) were analyzed using fastGWA-GLMM software [5].

### Analysis with two-sample MR and bi-directional MR

The Wald ratio was used to estimate the effect size when there was only one SNP available as an instrumental variable. The inverse-variance weighted (IVW) was used as the main method for causal estimation when there were multiple SNPs used as proxies. In addition, we used MR-Egger and weighted median methods for sensitivity analyses. The MR-Egger method was used to identify potential pleiotropy effects based on the *p*-value for the intercept. The weighted median method is utilized to strengthen causal estimates. We also performed bi-directional MR using cholelithiasis as the exposures and campesterol from CLSA cohort as the outcome. We selected IVs using the conventional genome-wide significance (*p* < 5 × 10^−8^) with linkage disequilibrium (LD) threshold of R2 < 0.001 within ± 5000 kilobase (kb) distance for the extraction of LD-independent SNPs from the corresponding GWAS used as instrumental variables.

### Colocalization analysis

We also performed a colocalization analysis between the hit (campesterol) and cholelithiasis with coloc R package (https://cran.r-project.org/web/packages/coloc/vignettes/a01_intro.html). The SNP would be considered as causal to one trait when the default prior probability was lower than 1 × 10^−4^. If the posterior probability of one SNP being shared between the two traits in one region was greater than 0.95, we considered it as a signal of colocalization. The Locus zoom plots were generated using the online website tool (http://locuszoom.org/) based on the GRCh17 reference genome.

### Statistical analysis and tools

The MR results are 2-sided, and a *p*-value < 0.05 was considered statistically significant. We apply multiple testing *p*-value corrections (FDR or Bonferroni) to reduce false-positive rates which we will described further in the results. All the analyses were performed on R platform (version 4.0.2). The “TwoSampleMR” (0.5.5), “Mendelian Randomization” (0.5.0), and “ggplot2” packages were used for statistical analyses and data visualizations.

## Results

**Fig. 1:**
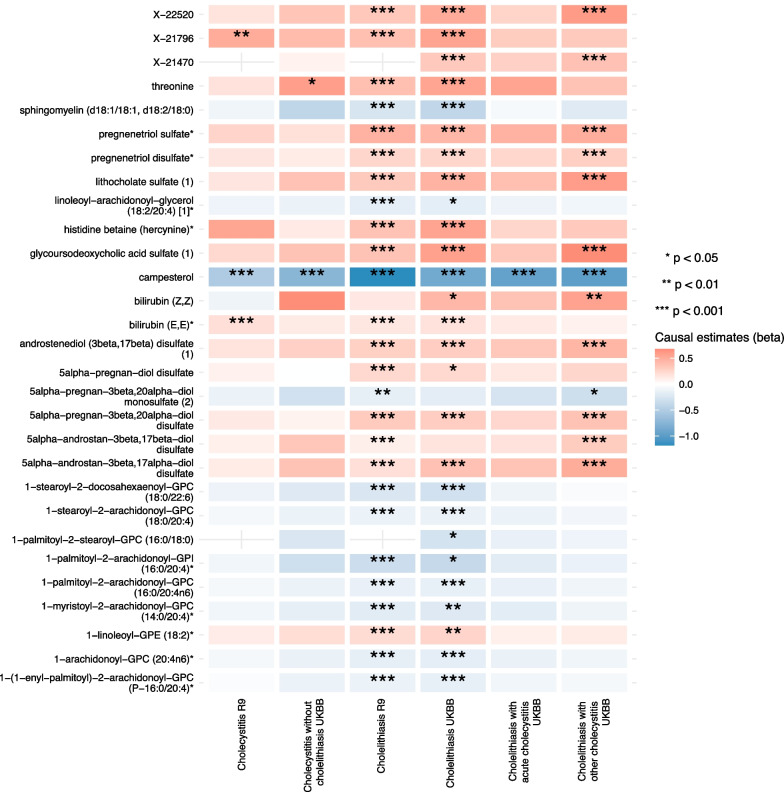
Heatmap showing the causal estimates of metabolites (with valid SNPs as instrumental variables ≤ 3) on the risk of cholelithiasis and cholecystitis using IVW fixed effect model. The *p*-value is adjusted using Bonferroni method. IVW, inverse-variance weighted; R9, FinnGen Release 9; UKBB, UK Biobank

In the univariable MR, we tested the association of each metabolic trait on the risk of the cholelithiasis and cholecystitis from different population cohorts. In order to obtain the best hit with least false positive probability, we set the adjusted *p*-value using Bonferroni method (for IVW [fixed-effect model], wald ratio, weighted-median), adjusted *p*-value using Benjamini–Hochberg method for IVW random-effect model and unadjusted *p*-value (for ME-egger). Interestingly, we observed consistent inverse correlation of campesterol on the risk of cholelithiasis and cholecystitis from both cohorts using IVW method, with the OR = 0.307 (95% confidence interval[CI] 0.294–0.321, *p*-value = 1.80 × 10^−14^ in cholelithiasis in FinnGen R9), OR = 0.598 (95% CI 0.530–0.676, *p*-value = 1.21 × 10^−16^ in cholecystitis in FinnGen R9), OR = 0.418 (95% CI 0.384–0.455, *p*-value = 3.35 × 10–89 in cholelithiasis in UK Biobank), OR = 0.479 (95% CI 0.416–0.552, *p*-value = 1.17 × 10^−24^ in cholecystitis without cholelithiasis in UK Biobank), OR = 0.388 (95% CI 0.323–0.467, *p*-value = 6.28 × 10^−24^ in cholelithiasis with acute cholecystitis in UK Biobank) and OR = 0.376 (95% CI 0.341–0.415, *p*-value = 5.01 × 10^−84^ in cholelithiasis with other cholecystitis in UK Biobank). Our sensitivity analyses using weighted-median and MR-egger methods also showed consistent trend when the tests were available indicating the robustness of the inverse correlation between the genetically predicted campesterol level and the risk of developing cholelithiasis and cholecystitis. The heatmap representation of causal estimates of metabolic traits with at least one statistical significance on the outcomes are shown in Fig. 1 and Additional files 1–4: Figs. S1–S4. The overall causal estimates were listed in Additional file 5: Tables S1–S7. The results of the pleiotropic and heterogeneity testing were shown in Additional file 5: Tables S8–S19. The statistical results of each SNP were shown in Additional file 5: Table 20–25.

In the bi-directional MR, we observed that cholelithiasis was positively associated with the plasma campesterol level, with the OR = 1.160 (95% CI 1.064–1.264, *p*-value = 7.130 × 10^−04^) as shown in IVW method. The sensitivity analyses using weighted-median (OR = 1.190, 95% CI 1.078–1.313, *p*-value = 5.838 × 10^−04^) and MR-egger (OR = 1.351, 95% CI 1.142–1.597, *p*-value = 6.554 × 10^−04^) method indicated consistent results (Additional file 5: Table 26–29). However, for cholecystitis, we only obtain 2 SNPs as proxies, the *p*-value for IVW fixed-effect model was 0.910 (95% CI 0.735–1.127, *p*-value = 0.389) with no statistical significance.

**Fig. 2:**
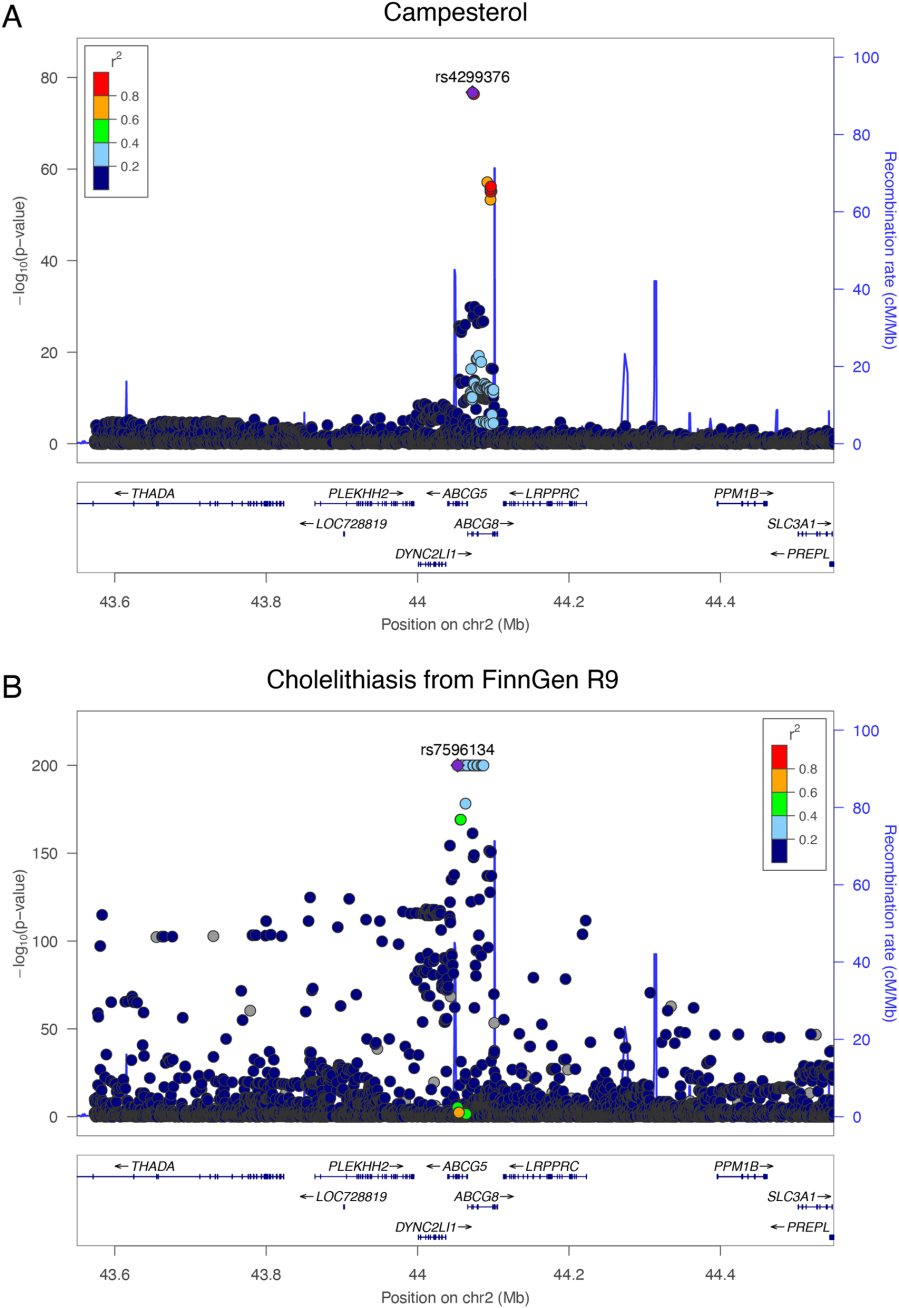
Colocalization analyses indicating the highlighted SNPs in campesterol (**A**) and cholelithiasis (**B**). The SNPs with significant *p*-value are located at the ABCG8 and ABCG5 loci. Notes: ABCG5, ATP Binding Cassette Subfamily G Member 5; ABCG8, ATP Binding Cassette Subfamily G Member 8

In colocalization analysis, we identified one candidate causal SNP rs4299376 is in region Chr2: 44,072,576, at *ABCG5*/*ABCG8* gene locus, with a posterior probability of 1. The *ABCG5*/*ABCG8* genes, encode membrane proteins responsible for plant sterol/cholesterol secretion to the gut lumen (bile duct and gut). We highlighted this region on both campesterol and cholelithiasis (FinnGen R9) using locus zoom shown in Fig. 2.

## Discussion

Campesterol, which belongs to plant sterols, is the analogue of cholesterol in nature. Plant sterol consists of a large category which cannot be synthesized endogenously by human body. Natural plant sterols mainly come from vegetable oils, nuts, grains and grain-derived products [6]. Previous studies showed that the plant sterol might be more hydrophobic, which can displace cholesterol from the micelles and limit the solubility of cholesterol and the hydrolytic process of cholesterol esters in the intestine [7]. Animal studies demonstrated that **i**ncidence of gallstone in mice was decreased in the group with plant sterol supplements. Furthermore, the serum cholesterol and intestinal cholesterol absorption would decrease as a result of downregulated cholesterol transport and metabolism in the liver.

However, the correlation between plant sterol and the incidence of cholelithiasis was still under debate. One study from the Finnish cohort suggested that early exposure to lower level of cholesterol and various types of plant sterols showed increased risk of developing gallstone disease. Also, in this study, the level of campesterol was considered as a surrogate marker for cholesterol absorption. It concluded that a lower serum cholestanol and plant sterol ratios during normal Western diet in the childhood may have predictive values for gallstones in the adulthood [8]. However, other studies have the competing perspective. One cross-sectional study indicated that relatively lower levels of serum plant sterol, lower ratios of plant sterol to cholesterol precursors, and higher levels of cholesterol precursors appear in gallstone patients, whereas biliary plant sterol and cholesterol concentrations were elevated in this population as compared with the controls.

Here, in this study, we used two-sample MR method to screen for metabolic factors that were causally associated with cholelithiasis and cholecystitis. Interestingly, we identified a consistent and strong inverse correlation between the genetically predisposed plasma campesterol level and the risk of cholelithiasis and cholecystitis. The bi-directional MR method indicated that the individuals with higher risk of developing cholelithiasis tend to have higher levels of campesterol in the blood. For the mechanistic studies, we performed the colocalization analyses and identified that the shared SNP was nearest to *ABCG5*/*ABCG8* gene. The *ABCG5* and *ABCG8* genes are sitting next to each other, and they encode protein products forming a heterodimer located on the cell membrane. These two genes have relatively higher expression in enterocytes and hepatocytes and are responsible for the transportation of the plant sterol and cholesterol from the blood stream to the bile duct (by hepatocytes) and gut lumen (by enterocytes) [9]. Our results suggested that the increased functionality of ABCG5/ABCG8 protein complex might increase the excretion of plant sterol/cholesterol into the bile duct and consequently lead to cholesterol stone formation and decrease the levels of plasma cholesterol and plant sterol are downregulated [3].

## Conclusion

Our comprehensive MR study reveal that the genetically predicted plasma campesterol level showed causal effects in reducing the risk of cholelithiasis and cholecystitis. Drug discovery targeting ABCG5/ABCG8 protein for the transportation of sterol/cholesterol body distribution might be a promising therapeutic strategy.

### Supplementary Information

**Additional file 1: Fig. S1** Heatmap showing the causal estimates of metabolites (with valid SNPs as instrumental variables > 3) on the risk of cholelithiasis and cholecystitis using IVW random effect model. The *p*-value is adjusted using Benjamini–Hochberg method (known as FDR). FDR, false discovery rate; R9, FinnGen Release 9; UKBB, UK Biobank

**Additional file 2: Fig. S2** Heatmap showing the causal estimates of metabolites (with only 1 SNP as valid instrumental variable) on the risk of cholelithiasis and cholecystitis. The *p*-value is adjusted using Bonferroni method. R9, FinnGen Release 9; UKBB, UK Biobank

**Additional file 3: Fig. S3** Heatmap showing the causal estimates of metabolites on the risk of cholelithiasis and cholecystitis using weighted-median model. The p-value is adjusted using Bonferroni method. R9, FinnGen Release 9; UKBB, UK Biobank )

**Additional file 4. Fig. S4** Heatmap showing the causal estimates of metabolites on the risk of cholelithiasis and cholecystitis using MR-egger method. The *p*-value is not adjusted for multiple testing. R9, FinnGen Release 9; UKBB, UK Biobank

**Additional file 5. Table S1–29** The calculated estimates, sensitivity analyses and heterogeneity results of the MR analyses.

## Data Availability

The summary-level results of cholelithiasis and cholecystitis datasets can be obtained from FinnGen Round 9 (https://r9.finngen.fi/). The summary-level results of cholelithiasis and cholecystitis from UK BioBank can be obtained from YangLab website (https://yanglab.westlake.edu.cn/data/ukb_fastgwa/imp/). The summary-level results of metabolic traits can be extracted from GWAS catalog website (https://www.ebi.ac.uk/gwas/home).
