# Supplementary figures and images for: Plasma campesterol and ABCG5/ABCG8 gene loci on the risk of cholelithiasis and cholecystitis: evidence from Mendelian randomization and colocalization analyses
Source: Hum Genomics. 2024 Feb 12;18:19. doi: 10.1186/s40246-024-00583-y (PMC10863091; doi:10.1186/s40246-024-00583-y)

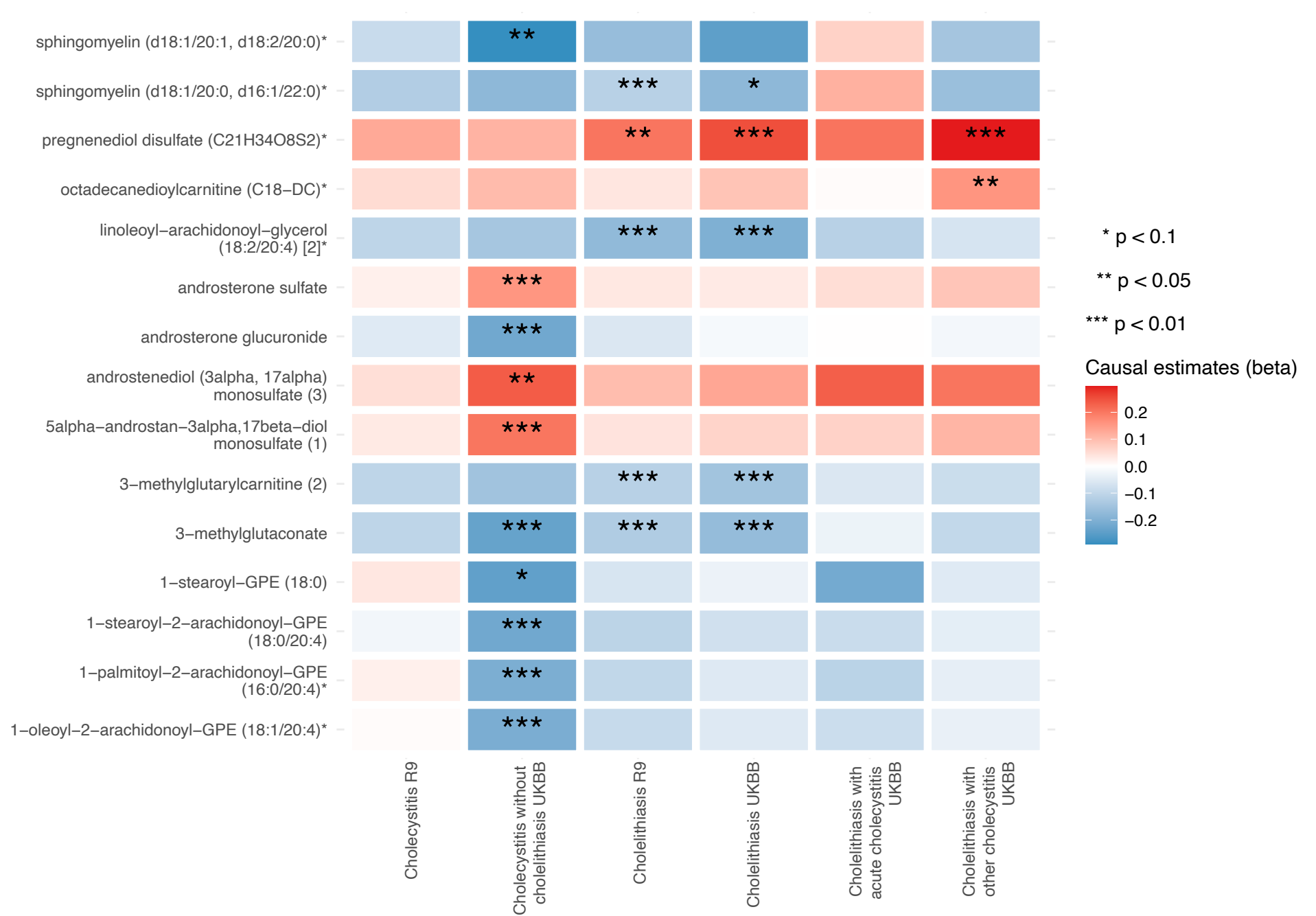

Supplement: Supplementary file 1 — Additional file 1: Fig. S1 Heatmap showing the causal estimates of metabolites (with valid SNPs as instrumental variables > 3) on the risk of cholelithiasis and cholecystitis using IVW random effect model. The p-value is adjusted using Benjamini–Hochberg method (known as FDR). FDR, false discovery rate; R9, FinnGen Release 9; UKBB, UK Biobank [file 40246_2024_583_MOESM1_ESM.pdf]

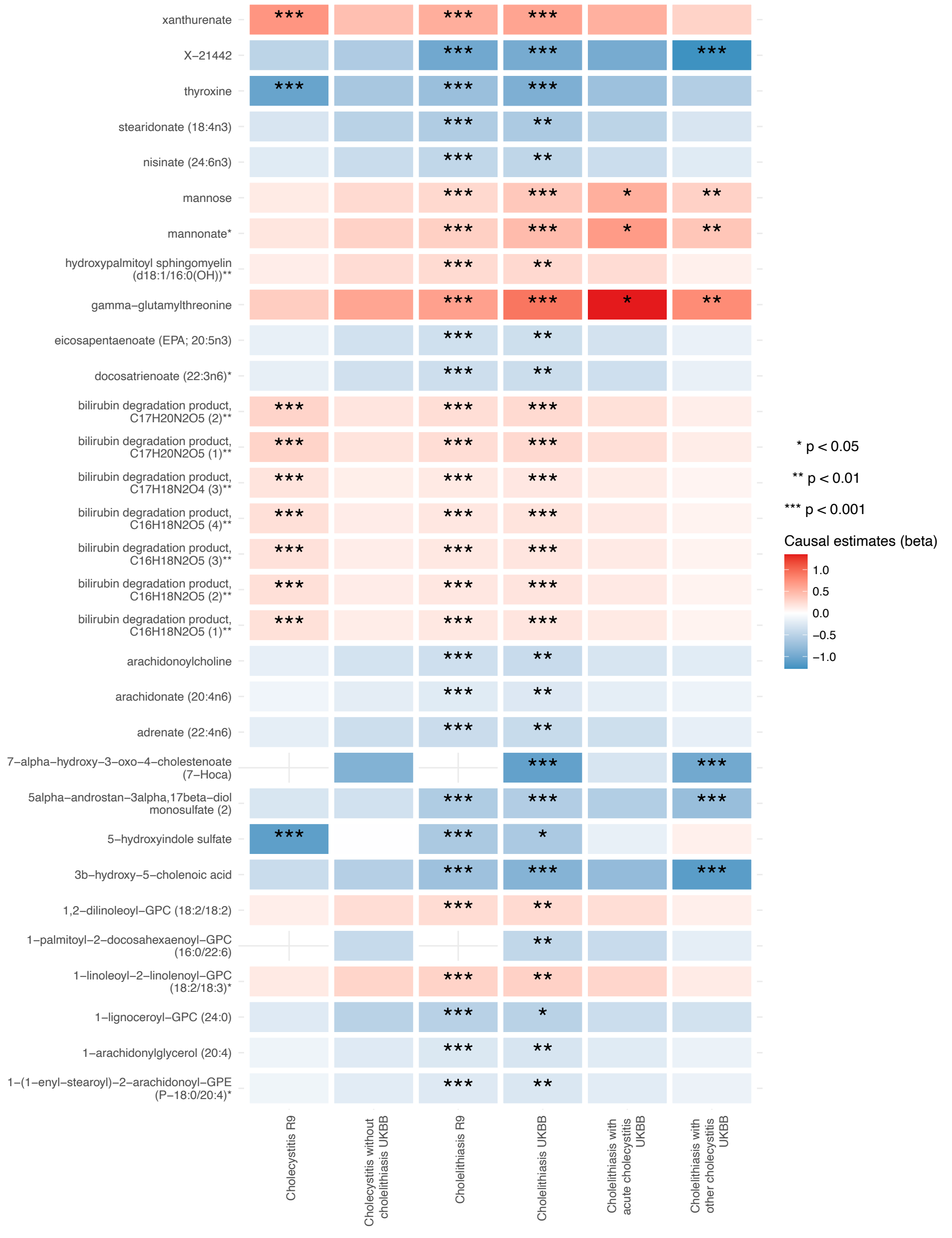

Supplement: Supplementary file 2 — Additional file 2: Fig. S2 Heatmap showing the causal estimates of metabolites (with only 1 SNP as valid instrumental variable) on the risk of cholelithiasis and cholecystitis. The p-value is adjusted using Bonferroni method. R9, FinnGen Release 9; UKBB, UK Biobank [file 40246_2024_583_MOESM2_ESM.pdf]

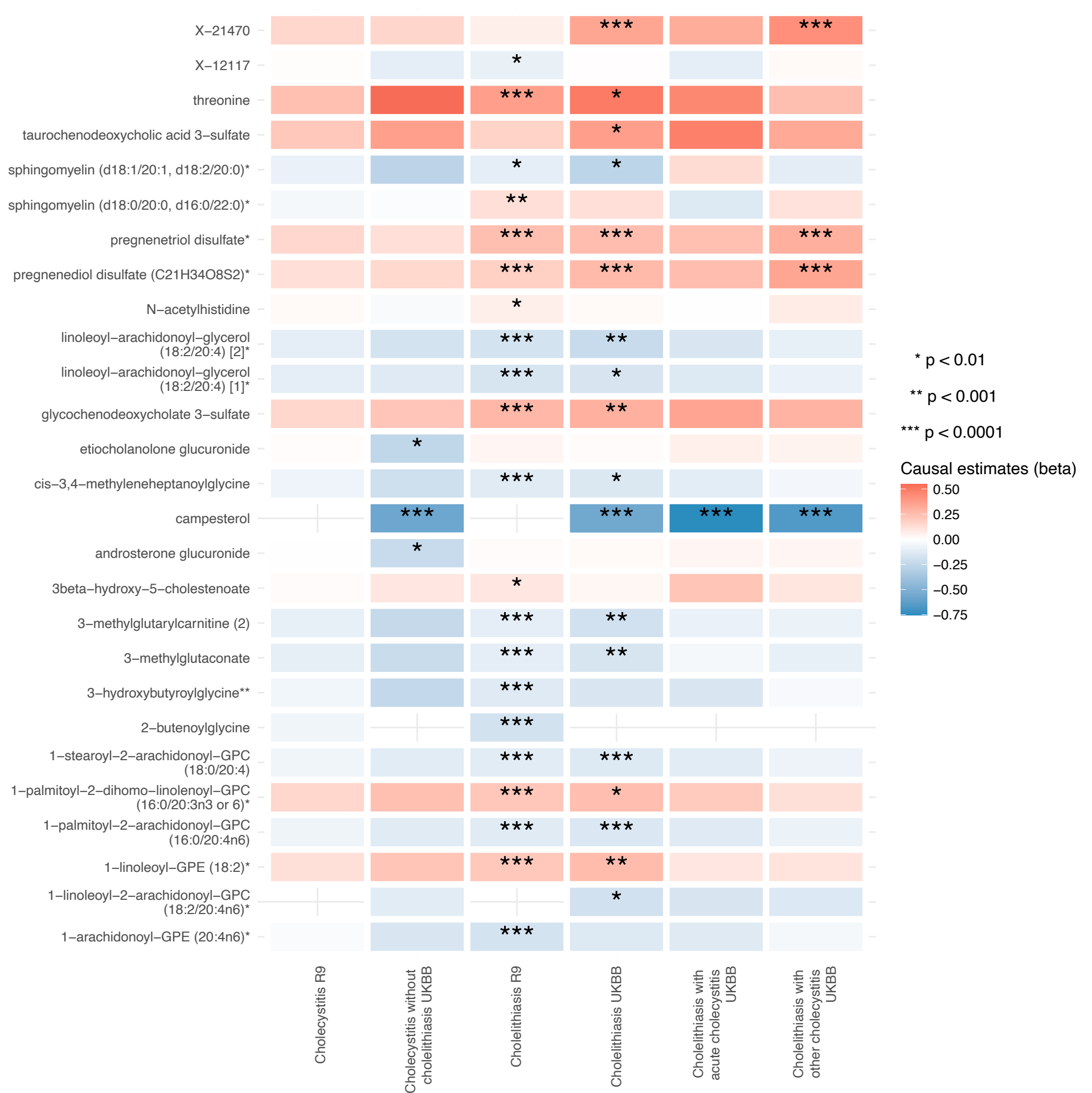

Supplement: Supplementary file 3 — Additional file 3: Fig. S3 Heatmap showing the causal estimates of metabolites on the risk of cholelithiasis and cholecystitis using weighted-median model. The p-value is adjusted using Bonferroni method. R9, FinnGen Release 9; UKBB, UK Biobank ) [file 40246_2024_583_MOESM3_ESM.pdf]

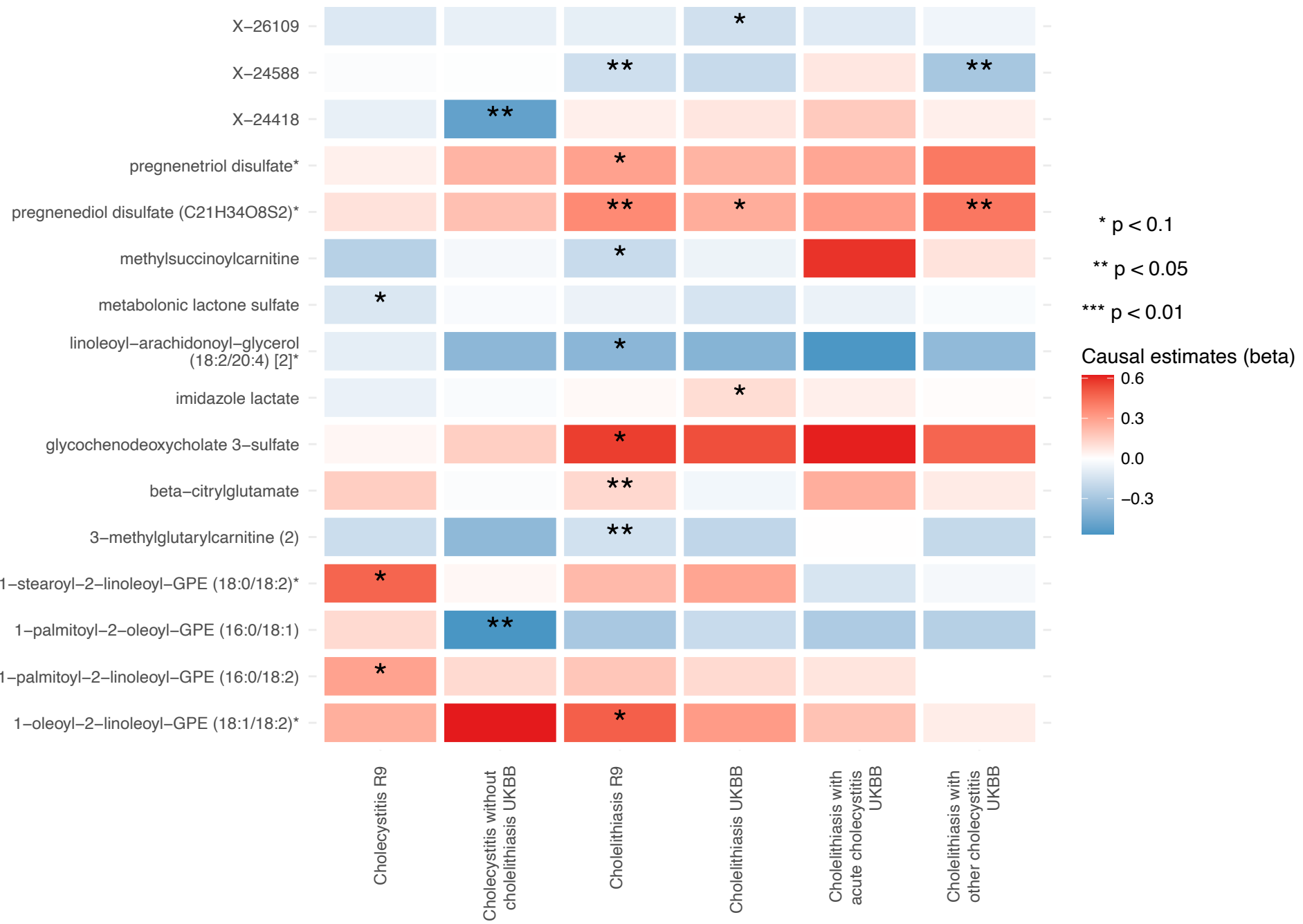

Supplement: Supplementary file 4 — Additional file 4. Fig. S4 Heatmap showing the causal estimates of metabolites on the risk of cholelithiasis and cholecystitis using MR-egger method. The p-value is not adjusted for multiple testing. R9, FinnGen Release 9; UKBB, UK Biobank [file 40246_2024_583_MOESM4_ESM.pdf]
